# Reproducible long-term cycling data of Al_2_O_3_ coated LiNi_0.70_Co_0.15_Mn_0.15_O_2_ cathodes for lithium-ion batteries

**DOI:** 10.1038/s41597-022-01217-5

**Published:** 2022-03-30

**Authors:** Rajendra S. Negi, Matthias T. Elm

**Affiliations:** 1 Center for Materials Research (LaMa) Justus-Liebig-University Giessen, Heinrich-Buff-Ring 16, D-35392 Giessen, Germany; 2 Institute of Physical Chemistry, Justus-Liebig-University Giessen, Heinrich-Buff-Ring 17, D-35392 Giessen, Germany; 3 Institute of Experimental Physics I, Justus Liebig University Giessen, Heinrich-Buff-Ring 16, 35392 Giessen, Germany

**Keywords:** Batteries, Batteries

## Abstract

LiNi_*x*_Co_*y*_Mn_1-*x*-*y*_O_2_ (NCM) based cathodes for Li-ion batteries (LIBs) are of great interest due to their higher energy density and lower costs compared to conventional LiCoO_2_ based cathodes. However, NCM based cathodes suffer from instabilities of the cathode-electrolyte interface resulting in faster capacity fading during long-term cycling. Different NCM compositions along with different coatings have been developed to protect the interface. However, a detailed understanding why and how coatings work is still missing. Up to now, no state-of-the-art NCM or coating material have been agreed upon yet, making it difficult to benchmark the performance of the coating material. Undefined standards complicate the use of experimentally produced data for model-based studies, which are a key element in assessing the beneficial effect of coatings. In this work, we therefore describe reproducible long-term cycling data of NCM based cathodes with and without an Al_2_O_3_ based coating. The data set is provided to be used for parameter fitting and/or as training data to encourage the simulation of the performance of LIBs in model-based approaches.

## Abstract (summary)

**Table:** 

Measurement(s)	specific discharge capacity • electrochemical-induced impedance spectroscopy
Technology Type(s)	battery cycler • potentiostat

## Background & Summary

Lithium-ion batteries (LIBs) are considered as state-of-the-art energy storage technology for various applications and have drawn extensive attention in the past decades^1,2^. They are currently powering a broad range of portable electronic devices (cameras, cell phones, power tools etc.), as well as hybrid and fully electric vehicles (EVs)^3,4^. However, further improvement in the properties of LIBs (achieving higher C-rate performance and more achievable capacity) is necessary to open up new applications along with expanding the market for the present ones. For example, shortening the charge time from hours to minutes can benefit the electric vehicle market to develop supercharge stations, while an increase of the practical charge capacity can extend the distance covered per charge^5^. However, in the conventional LIB configuration the cathode material LiCoO_2_ is a limiting factor for achieving maximum capacity due to its low lithium utilization (i.e., < 60%) and its relatively low specific capacity (150 mA h g^−1^)^6,7^. Several efforts have been made in the last two decades to develop new cathode materials. Among all NCM based cathode stand out the most^8^.

Nickel-rich LiNi_*x*_Co_*y*_Mn_1-*x*-*y*_O_2_ (NCM) based cathode materials are consider as the benchmark for advanced lithium-ion batteries (LIBs) due to their high energy and power density along with lower costs compared to conventional LiCoO_2_ cathodes. These advantages make NCM based cathodes ideal candidates for long range electro-mobility application^7,9^. It is proven successfully that increasing the Ni content increases the achievable capacity. Simultaneously, an increase of the Mn content improves the structural stability of layered NCM cathodes^9^. However, these NCM based cathodes still show instability issues due to transition metal dissolution, surface reconstruction, electrolyte decomposition, and surface reactions during charge/discharge cycles^7,9^.

Considerable amount of effort has been dedicated to modify the surface of NCM based cathodes (via coating) in order to quarantine the bulk material from the electrolyte, thus, decreasing the NCM surface reactivity with the electrolyte to prevent surface reconstruction^10,11^. Several oxide based coating materials have shown to be effective against the instability of the NCM surface^12–14^. Among all, Al_2_O_3_ has been considered as one of the most promising coating material for NCM based cathodes in LIBs due to its low cost and rather simple coating procedures^15–19^. However, a detailed understanding how the coating works and how it affects the long-term cycling is still missing.

Model-based approaches in predicting the cell performance as well as the battery cycle life are one of the key elements in optimizing the coating and, thus, in a further development of LIBs^20–26^. Here, reliable datasets of the cell performance are needed as input parameters or training data for data-driven modelling. Thus, the main focus of the work is to provide data of the performance of LIBs with pristine and coated NCM cathodes. The coating is obtained by applying a recently developed coating method for Al_2_O_3_. In our recent work^18^, we demonstrate that the Al_2_O_3_ coating significantly improves the long-term cycling stability of NCM cathodes, as the coating prevents surface degradation as well as cracking of the NCM particles during cycling. In our recent publication, we present the averaged data of three independent cells. The data provided here are the corresponding experimentally reproducible data for long term cycling (i.e. the repeated charge/discharge,) of the three cells for the two different kinds of NCM cathodes each (pristine and coated). In addition, long term cycling data of a fourth cell (pristine and coated) are provided, which are not discussed in our recent publication. The impedance data after the 1^st^ and 100^th^ cycle including the corresponding fits are also provided, which confirm that the coating effectively impedes the formation of a resistive cathode solid−electrolyte interphase. In addition, we give a more detailed description of the measurement procedures reported in our recent work^18^ to ensure that the results can be reproduced by other groups. The reproducibility of data and a detailed description of the measurement procedure is mandatory for the use in model-based studies. Thus, the data provided in the work are considered to be reused for parameter fitting or as training data in model-based studies along with the new coating method for different cathode materials. In addition, the data can be used to support the design and the evaluation of the effect of different coating methods on NCM based cathodes in LIBs.

## Methods

### Preparation of surface coating

The Al_2_O_3_ based coating is prepared via a wet-chemical route. The whole coating process is performed in a glovebox. Initially, trimethylaluminium solution (TMA, 0.279 mL, 2 M in toluene; Sigma-Aldrich) is diluted by adding anhydrous toluene (15 mL, 99.8%; Sigma-Aldrich). The resulting solution is stirred on a magnetic stirrer for 1 h in order to get a homogenous mixture. For the coating process, the diluted TMA solution (5 ml) is added to LiNi_0.70_Co_0.15_Mn_0.15_O_2_ (NCM701515, 5 g; Gelon LIB) powder in a small glass flask and the mixture is stirred at 30 °C for 12 h. The final dispersion product is filtered and washed using anhydrous toluene (20 mL, 4 times 5 mL). The recovered coated NCM powder is further dried in vacuum (200 °C) for 12 h and then finally transferred to the glovebox. The pristine powder is named P-NCM and the coated powder is named C-NCM in the following. These methods are expanded versions of descriptions in our related work.

### Electrode preparation

At first, 5 wt% solution of polyvinylidenefluoride binder (Solef PVDF 6020; Solvay) is prepared in N-methyl-2-pyrrolidone (NMP; Sigma-Aldrich) solution. 95 mg of NMP solution is added to 5 mg of PVDF in a glass flask and stirred for 12 h at 60 °C on a magnetic stirrer. For the electrode preparation, P-/C-NCM powder (90 wt%) is mixed with conductive carbon black (5 wt%, Super P; TIMCAL) and 5 wt% of PVDF solution is added. Subsequently, additional NMP is added in order to control the viscosity of the mixture and the solution is stirred for 12 h. The resulting mixture is casted on Al foil using a doctor blade and dried afterwards. Finally, the as-prepared NCM casted sheets are calendared at 90 psi (no hot rolling press is used), dried at 120 °C under vacuum, and then transferred to the glovebox for further studies.

### Assembly of coin cells

**Fig. 1:**
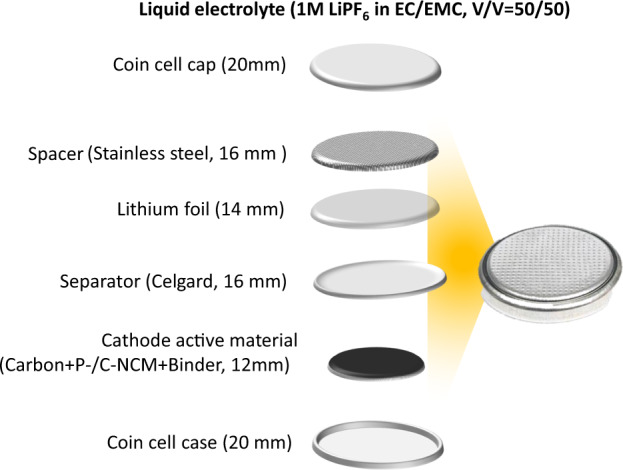
Schematic of the cell assembly of the LIBs coin-type cell.

For the cell preparation, coin cells (CR2032; MTI Corporation) are prepared using P-/C-NCM as cathodes (12 mm) and Li foil as anode (14 mm, Rockwood Lithium GmbH). At first, the NCM cathodes are put on the coin cell case and a Celgard separator (16 mm; Celgard2500) is put on the top. Then, 50 μL of LP50 (1 M LiPF_6_ in 1/1 wt/wt ethylene carbonate/ethyl methyl carbonate; Sigma-Aldrich) electrolyte is added and the Li foil is put on the top along with a stainless steel spacer. The whole assembly is then closed using a second coin cell case. Figure 1 shows the information regarding the coin-cell type used along with the components. Table 1 lists the components used in this study. The prepared cells were given a rest time of 3 h before starting the electrochemical measurements.

**Table 1:** List of components used in the coin-type cell assembly of the battery.

Components	Material/Parameters
Cathode	P-/C-NCM (90 wt%) + Carbon (5 wt%) + PVDF (5 wt%) (12 mm)
Anode	Li foil (14 mm)
Separator	Celgard2500 (1 6 mm)
Electrolyte	1 M LiPF_6_ in 1/1 wt/wt ethylene carbonate/ethyl methyl carbonate (50 μL)
Spacer	Stainless steel (16 mm)
Coin-cell	CR2032

### Measurement and data collection

**Fig. 2:**
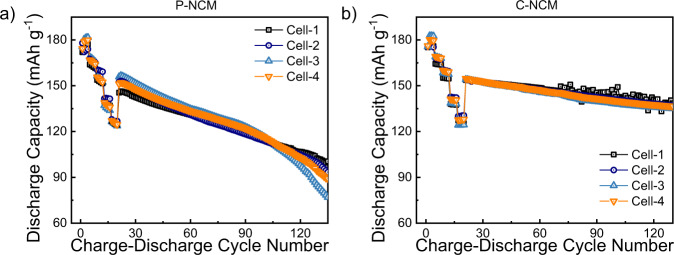
Reproducible long-term cycling performance of different cells: (**a**) P-NCM and (**b**) C-NCM. The data of cell 1, 2, and 3 are reproduced with permission from ref. ^18^.

**Fig. 3:**
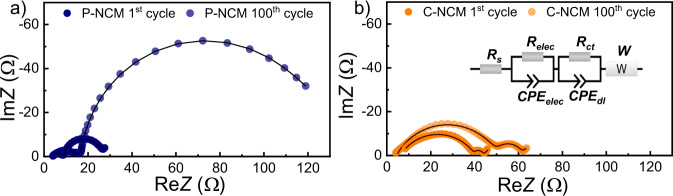
Nyquist plots of the electrochemical impedance measured in the fully charged state (4.3 V) for (**a**) P-NCM and (**b**) NCM after the 1^st^ and 100^th^ cycle. Reproduced with permission from ref. ^18^.

**Fig. 4:**
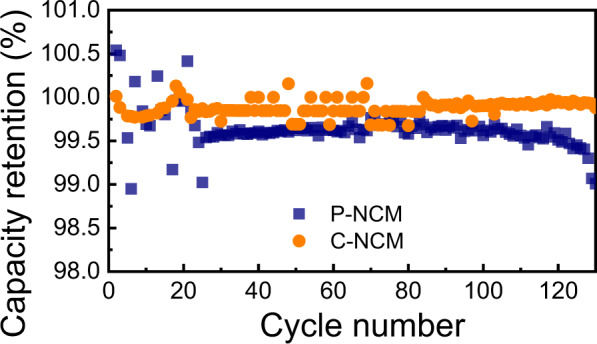
Comparison of the capacity retention for the P-NCM and C-NCM (cell 3).

All the electrochemical tests have been performed using a VMP-300 potentiostat/galvanostat (Biologic) and a BCS-805 battery cycler (Biologic) at room temperature (25 °C). For the cycling results, the coin cells have been cycled with a BCS-805 battery cycler (Biologic) using a constant current (CC) mode in the voltage range between 3.0 and 4.3 V. The 1 C capacity is chosen as 160 mA g^−1^ ^18^ For the measurements, the cells have been initially tested for rate-capability using different C-rate steps (0.1 C to 2 C, each for 4 cycles) in the program. For long term cycling the same cells have been cycled at 0.5 C for 135 cycles. For the calculation of the specific capacities and the specific currents, only the mass of the active material (NCM) is considered, which was determined for each cell. The absolute value of the specific current varies for each sample due to small differences in the active mass of the cells. Thus, only the C-rate is presented. For data reproducibility, four different cells of the same cathodes (P-/C-NCM) have been tested under the same conditions. The results are shown in Fig. 2. For the electrochemical impedance spectroscopy (EIS) measurement, a VMP-300 potentiostat/galvanostat (Biologic) has been used. EIS measurements have been performed on the cycled coin cells after the 1^st^ and 100^th^ cycle in fully charged state (4.3 V) in the frequency range between 100 kHz to 10 mHz. The results are shown in Fig. 3, revealing the beneficial effect of the coating. From the discharge capacities of the cells, the capacity retention has been calculated by dividing the discharge capacity of cycle (*n* + 1) by the discharge capacity of cycle *n*. A comparison of the capacity retention of pristine and coated NCM701515 (cell 3) is exemplary shown in Fig. 4.

## Data Records

The recorded data for all the experiments are provided as text files. The data can be divided into three categories: discharge profiles of the battery during C-rate and long-term cycling for eight different cells (4 for P-NCM and 4 for C-NCM) at various current densities (Fig. 2), the EIS measurements before and after cycling for both P- and C-NCM (Fig. 3), and the calculated capacity retention for all cells investigated (Fig. 4). The output files contain various information, including discharge capacity (mAh g^−1^), cycle number, impedance (Ω), frequency (Hz), and capacity retention (%), respectively. The datasets are available at the Open Science Framework (OSF) repository^27^. In Table 2, metadata for each descripted data column are presented.

**Table 2:** Metadata of cycle number, discharge capacity, impedance response, and capacity retention of the tested coin cells.

Value	Unit	Description
Cycle number	—	Number of respective discharge cycles have been labelled as *cycle number*
Capacity	mAh g^−1^	Measured capacity of the coin cells; labelled as *Discharge capacity*
Impedance	Ω	Measured impedance before and after cycling; labelled as *ReZ* (real part) and *ImZ* (imaginary part)
Capacity retention	%	Calculated capacity retention; labelled as *capacity retention*

## Technical Validation

For each experiment, a newly assembled coin cell with fresh cathode, anode and electrolyte has been used. However, slight deviations in the battery capacity are apparent and this inconsistency probably arises from unavoidable assembling error (such as the position of electrodes inside coin cell). Even though the deviations also influence the initial capacity, the impact on the coin-cell capacity is more apparent during long-term cycling. To evaluate the reproducibility of the cells, four different coin cells with the same batch of cathode have been prepared and tested. Comparing all the cells reveals that there are only small differences in the capacity values of the different cells, confirming the good experimental reproducibility along with the beneficial effect of the coating.

## Usage Notes

The data provided herein are considered to be used in validating model-based studies related to Li-ion batteries. It is worth noting that only the cycling data of the first 135 cycles can be provided, which limits a reliable prediction of the failure of the cells, in particular, for the coated ones. Nevertheless, we encourage to use the cycling data to obtain fit parameters (e.g. cycling potential window, cycling conditions etc.), which are needed for empirical model systems. In addition, the data can be used in model-based studies to correlate the coating properties (such as thickness, homogeneity) with the cathode cycling behavior to assess the influence of coating on the electrochemical performance. This will help to develop new coating strategies for improved LIBs.

## Data Availability

The reported data were generated from experiments. No custom code was used to generate or process the data.
